# Comprehensive Evaluation of the Sustainability of Waste Concrete towards Structural Concrete Application in Freeze-Thaw Environment

**DOI:** 10.3390/ma15176153

**Published:** 2022-09-05

**Authors:** Da Wei, Pinghua Zhu, Shan Gao, Xiancui Yan, Hui Liu, Haifeng Fan

**Affiliations:** 1 Department of Civil Engineering, Changzhou University, 21 Gehu Middle Road, Wujin District, Changzhou 213164, China; 2 School of Civil Engineering, Harbin Institute of Technology, Harbin 150090, China; 3 State Key Laboratory of Silicate Materials for Architectures, Wuhan University of Technology, Wuhan 430070, China

**Keywords:** freeze-thaw damage, parent concrete, structural concrete, recycled aggregate concrete, performance, sustainability

## Abstract

To promote the in-situ and structural application of waste concrete in cold regions, the sustainable application potential of waste concrete in a freeze-thaw (F-T) environment was comprehensively evaluated from three aspects of performance, environmental load, and economic benefit. The recycled aggregate concrete (RAC) was produced by recycled coarse aggregate (RCA), which was obtained from the crushing of natural aggregate concrete (NAC) after every F-T 150 cycles until F-T failure. The effects of F-T damage of parent concrete on the physical properties of RCA and mechanical and frost resistance of RAC under 35% flexural stress were studied. Besides, the sustainability of NAC and RAC was compared and analyzed by emergy theory. The results suggested that the physical properties of RCA deteriorated gradually with the accumulation of F-T damage to parent concrete. The RCA obtained from parent concrete that suffered F-T damage could be used as coarse aggregate for structural concrete when F-T damage is smaller than 0.367. The F-T damage of parent concrete had an adverse effect on the mechanical properties and frost resistance of RAC. The frost resistance of RAC obtained from parent concrete with larger F-T damage was worse. The RAC prepared from parent concrete without F-T failure can serve 50 years in cold regions, while that with F-T failure can only serve 30 years. The F-T damage microelements were dispersed in the adhesive mortar of RCA and transferred to RAC, resulting in the reduction of the mechanical properties and frost resistance of RAC. Emergy analysis showed that the reuse of waste concrete after F-T failure required higher economic input, higher environment load, lower output efficiency, and sustainability. The performance, environmental load and economic benefit of RAC prepared by using waste concrete after F-T failure were inferior to that of waste concrete without F-T failure. Waste concrete after F-T failure is not recommended to be used as coarse aggregate for structural concrete.

## 1. Introduction

The adoption of sustainable consumption and production patterns is one of the key goals of the United Nations 2030 Agenda for Sustainable Development. At present, in order to achieve socially sustainable production and promote the development of a green economy, the recycling and utilization of materials have been paid attention to in many fields, such as medical treatment, food processing, bioenergy, electronic equipment, and construction [1,2,3,4,5].

The construction industry plays an important role in carbon emissions, and its sustainable production patterns are always being explored. Using waste concrete to produce recycled coarse aggregate (RCA) and reuse it in structural concrete can facilitate sustainable development and obtain remarkable economic effectiveness. Meanwhile, it is of great significance for the achievement of United Nations Sustainable Development Goals such as protecting terrestrial ecology, reducing carbon emissions, and coping with climate change. However, due to the different concrete service environments and cementitious materials compositions all over the world, the properties of RCA are different [6]. The addition of industrial wastes such as fly ash and slag replaced part of cement to meet the principle of preventing pollution at the source, low energy consumption, and using renewable raw materials in green chemistry. The physical properties of RCA are significant for the reuse of waste concrete. Therefore, many countries have formulated RCA specifications in structural concrete to promote the use of local waste concrete [7].

In the reusing process, natural aggregate concrete (NAC) is generally as the parent concrete. The compressive strength of parent concrete affects the content and quality of adhesive mortar of RCA [8], which lead to differences in the physical properties of RCA. Liu et al. [9] found that higher strength of parent concrete led to better physical properties of RCA. Kou and Poon [10] considered that with the decrease of the compressive strength of parent concrete, the water absorption of RCA under the same particle size increased, while the apparent density decreased. Nevertheless, research on the physical properties of RCA produced from parent concrete exposed to the actual service environment is limited.

The performance of parent concrete also has a significant influence on the mechanical properties and durability of recycled aggregate concrete (RAC) [11]. Ahmad Bhat et al. [12] compared the mechanical properties of RAC produced by parent concrete with a compressive strength between 20 MPa and 60 MPa, and found that the mechanical properties of RAC prepared by higher compressive strength parent concrete were higher under the same RCA replacement. Zhang et al. [13] believed that RCA from low-strength parent concrete cannot produce medium-strength or high-strength RAC. In terms of durability, Mi et al. [14] pointed out that the carbonation resistance of RAC can be increased by improving the compressive strength ratio between parent concrete and RAC. Both Kou and Poon [10] and Ying et al. [15] found that RAC produced by high-strength parent concrete had better chloride ion penetration resistance. Liu et al. [9] believed that the RAC from high-strength or air-entrained parent concrete had excellent frost resistance. Many studies have been carried out on the effect of the strength of parent concrete on the performance of RAC, while limited works were performed on the recycling potential of parent concrete after actual service life.

In cold regions, a large number of hydraulic structures are exposed to a freeze-thaw (F-T) environment, such as dams and bridges. The frost resistance of concrete becomes an important durability index to evaluate the health of structures. Once F-T destruction of concrete occurred, it will become waste concrete because of the loss of bearing capacity and protection ability to reinforcement [16]. Due to safety considerations, this kind of concrete was unable to recycle; its disposal problems cause serious economic loss and environmental burden [17]. To solve this problem, it is urgent to study the recycling potential of parent concrete with different F-T damage in the F-T environment.

At present, the frost resistance of RAC has been fully studied. The quality of RCA [18], replacement rate [19], water-cement ratio [20], and air entrainment [21] of RAC have major effects on frost resistance of RAC. However, the current research does not actually consider the source of RCA, that is, the actual service environment of parent concrete. RAC meets the service and environmental requirements of parent concrete, which can effectively solve the environmental and economic problems caused by space-time effects [22]. The key to solving this problem is whether RAC can achieve the ideal service life in the specific service environment of parent concrete. For the parent concrete from the F-T environment, the produced RAC is most likely to be reused in the F-T environment. Therefore, the research on the influence of F-T damage of parent concrete on the mechanical and frost resistance of RAC has important theoretical guiding significance for the engineering application in cold regions. Meanwhile, the environmental burden and economic benefit brought by RAC production were the important factors that restrict its application potential. As a consequence, it should not be limited to a single study of RAC performance, but should be combined with a comprehensive evaluation of performance, environmental load, and economic benefits [5].

Emergy analysis is an effective method to evaluate the sustainability of renewable resource recovery systems [23]. Based on natural value, this method converts various natural and social economic resources into solar energy values for research, and converts materials, energy, and information of different qualities into a dimensionally unified energy value—solar joules, thus realizing the docking of ecological flow and economic flow [24]. Yuan et al. [25] pointed out the closed-loop recycling of construction waste was better than open-loop recycling from three aspects of society, environment, and sustainability based on the emergy theory. Zhao et al. [26] believed that the emergy sustainability index (ESI) value of circular economy concrete production mode was greater than that of traditional NAC, and the circular economy concrete production was more sustainable. Introducing emergy theory into RAC production in the F-T environment will provide theoretical support for the application of RAC and sustainable development of the construction industry.

The purpose of this paper is to comprehensively evaluate the sustainable application potential of waste concrete in the F-T environment. C40 NAC was prepared as parent concrete. Under the simulated load-(F-T) coupling action, the parent concrete was crushed into RCA every F-T 150 cycles for the preparation of RAC until F-T failure. The effects of F-T damage on particle size and physical properties of RCA were analyzed. The F-T damage threshold of parent concrete was obtained based on the requirements of structural concrete aggregate. Besides, the effects of F-T damage of parent concrete on the mechanical properties and frost resistance of RAC were further studied, and the maximum service life of RAC in cold regions was obtained. Through the analysis of porosity and scanning electron microscope (SEM), the micro mechanism of the effect of parent concrete F-T damage on the properties of RCA and RAC was revealed. In addition, according to the emergy analysis theory, the emergy investment ratio (EIR), emergy yield ratio (EYR), environmental load ratio (ELR) and emergy sustainability index (ESI) of NAC and RAC were calculated, so that the sustainable utilization potential of waste concrete from three aspects of performance, environmental load and economic benefits were evaluated comprehensively.

## 2. Experimental

### 2.1. Materials and Preparation of Parent Concrete

Natural gravel was regarded as the natural coarse aggregate (NCA) to produce parent concrete. The physical properties of NCA were tested according to GB/T 14685(2011) [27], and the results are shown in Table 1. Natural river sand (0.15–4.75 mm) with a fineness modulus of 2.6 was regarded as the natural fine aggregate. Ordinary Portland cement with strength grade of 42.5, silica fume (SF), fly ash (FA) and slag (SL) were regarded as the binding materials, and their apparent densities were 3080, 2720, 2520, 2870 kg/m^3^ respectively. Besides, polycarboxylic acid (PCA) superplasticizer and GYQ-E100 air entraining agent (AEA) were also added to enhance the properties of concrete. The dosage of PCA and AEA is 0.5% and 0.06% of the quality of binding materials.

The strength grade of NAC was C40 and the target slump was 180 mm, which can satisfy the requirements of most projects. The ‘overall calculation method’ [23] was used to optimize the mix proportion design to ensure the excellent durability of concrete. This method established a universal volume model for concrete and had good engineering applicability [28]. The mix proportions are shown in Table 2. Two-stage mixing method was used for concrete mixing [29]. Specimens with size of 100 × 100 × 100 mm were used for compressive strength (*f_c_*) test. Specimens with size of 100 × 100 × 400 mm were used for flexural strength (*f_f_*) test and rapid F-T test. The specimens were placed in the standard curing chamber (the temperature of 20 ± 2 °C and relative humidity > 95%) for 28 days. The slumps of fresh concrete were determined according to GB/T 50080(2016) [30], and the results were also shown in Table 2.

### 2.2. The Load and Rapid F-T Coupling Test of Parent Concrete

**Figure 1:**
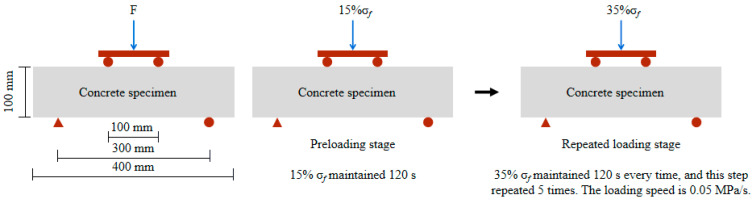
Loading procedure diagram.

**Figure 2:**
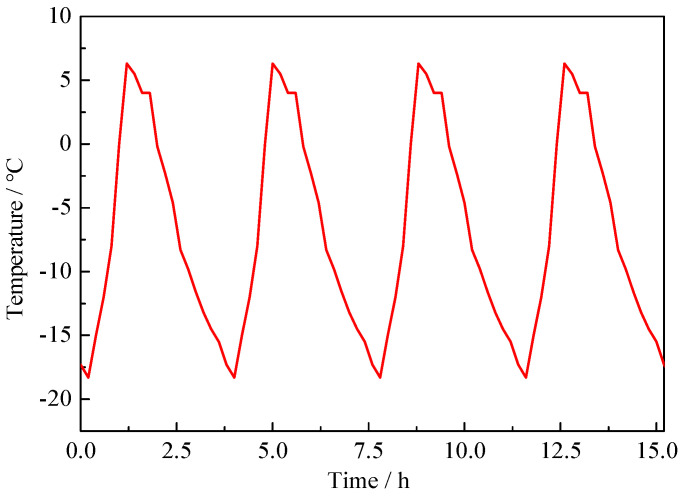
Temperature variation during F-T cycles.

The new rapid F-T test method of simulating load and F-T coupling test was adopted [31]. The flexural stress level used in the test is 35% ultimate flexural strength (σ*_f_*). Under this stress condition, the microcracks are stable. The specific loading procedure is shown in Figure 1. The specific application process is as follows:

The new method avoids stress loss due to the violent fluctuation of the rapid F-T test temperature. The mechanical properties and rapid F-T test of NAC were carried out according to GB/T 50081(2019) [32] and GB/T 50082(2009) [33] respectively. The temperature variation is shown in Figure 2, the temperature range is −18 ± 2~5 ± 2 °C. The mass loss rate (MLR) and relative dynamic modulus of elasticity (RDME) were tested every F-T 50 cycles, the calculation formulas are shown in Formulas (1) and (2) respectively. The *f_c_* and *f_f_* were tested every F-T 150 cycles.

(1) $$MLR=\frac{W_{0}-W_{i}}{W_{0}}\times 100\%$$

where, *W*_0_ represents the mass of concrete before F-T cycle; *W_i_* represents the mass of concrete after F-T *i* cycles.

(2) $$RDME=\left(\frac{f_{i}^{2}}{f_{0}^{2}}\right)\times 100\%$$

where, *f*_0_ represents the transversal fundamental frequency of concrete before F-T cycle; *f_i_* represents the transversal fundamental frequency of concrete after F-T *i* cycles.

### 2.3. Production of RCA

Five kinds of RCA from different F-T cycles of NAC were obtained. The NAC after 0, 150, 300, 450 and 600 F-T cycles were put into an oven with a temperature of 60 °C and dried for 24 h before crushing. To minimize the external load impact of crushing on RCA properties, NAC was crushed in jaw crusher twice. The outlet width of jaw crusher was set as 31.5 mm to control the particle size range (5–25 mm) of RCA. After cleaning and screening, RCA with particle size greater than 5 mm was obtained. The apparent density, water absorption, and crushing values of RCA were determined according to GB/T 25177(2010) [34]. The adhesive mortar content of RCA was calculated by high-temperature cycle method [35]. The physical properties of RCAs are also shown in Table 1.

### 2.4. Preparation of RAC

Five kinds of RAC were prepared and the corresponding relationships among NAC, RCA and RAC are shown in Table 3. The types of fine aggregate, cementitious materials, admixtures, mix design and the curing method of RAC were all the same as those of NAC. The mix proportions and slumps of RAC are also shown in Table 2.

### 2.5. The Rapid F-T Test of RAC

After 28 days of curing, the rapid F-T test was conducted on RAC to access the frost resistance. The load and rapid F-T coupling test method were consistent with that of parent concrete. The frost resistance of RAC was evaluated in terms of the *f_c_*, *f_f_*, MLR and RDME. The *f_c_* and *f_f_* were tested every 100 F-T cycles. The MLR and RDME were tested every 50 F-T cycles.

### 2.6. SEM and Porosity Test of RAC

The micromorphology of NAC and RAC after F-T cycles was observed with a JSM-IT100 scanning electronic microscopy instrument. The interfacial transition zone (ITZ) and pore changes of aggregate were observed. The change of porosity of RAC was measured by the ‘evaporable water content method’ [36]. The formula is shown in Formula (3).

(3) $$P=\frac{\left(m_{0}-m_{1}\right)\rho_{c}}{m_{0}\rho_{w}}\times 100\%$$

where, *P* is the porosity; *m*_0_ is the mass of RAC saturated by water absorption in vacuum; *m*_1_ is the constant mass of RAC under 105 °C; *ρ_c_* and *ρ_w_* are the densities of RAC and water respectively.

**Figure 3:**
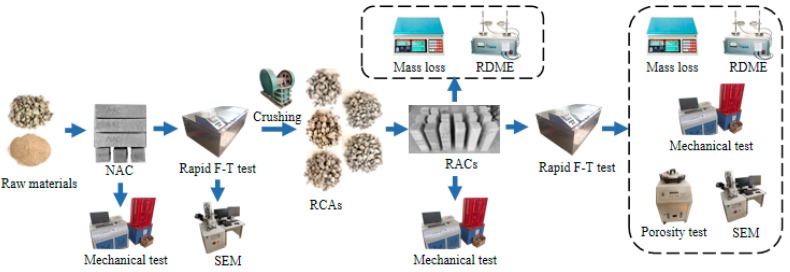
Experimental process.

Three specimens were prepared for each group of concrete to obtain the average value of three data calculations as the group result, and calculate the standard deviation as the error. The experimental process is illustrated in Figure 3.

### 2.7. Sustainability Analysis Method

Emergy analysis was used to analyze the sustainability of the RAC production mode. The emergy indexes involved in this study include: EIR, EYR, ELR and ESI. EIR reflected the level of economic input. The larger the EIR value of the production system, the higher the economic input required by the production system. EYR can reflect the output efficiency of the production system dynamically, and the larger the EYR value, the greater the production efficiency. ELR was used to evaluate the environmental burden caused by the production process. The more environmental burden caused, the greater the ELR value will be. Besides, ESI can evaluate the sustainability of production systems. The production system was sustainable and had great development potential when 1 ≤ ESI ≤ 10 [26]. The calculation formulas are as follows:

(4) $$E=\lambda E_{a}$$

where, *E* is emergy (sej); *λ* is emergy conversion rate (sej/J), which is the amount of emergy needed to produce one joule of product; *E_a_* is available energy (J).

(5) $$EIR=\frac{F}{N+R}$$

(6) $$EYR=\frac{Y}{F}$$

(7) $$ELR=\frac{F+N}{R}$$

(8) $$ESI=\frac{EYR}{ELR}$$

where, *F* is the emergy value of non-resource investment; *Y* is the emergy value of the product produced; *N* is the emergy value of non-renewable resources; *R* is the emergy value of renewable resources.

## 3. Results and Discussion

### 3.1. Performance of NAC during F-T Cycles

#### 3.1.1. Mechanical Properties of NAC

**Figure 4:**
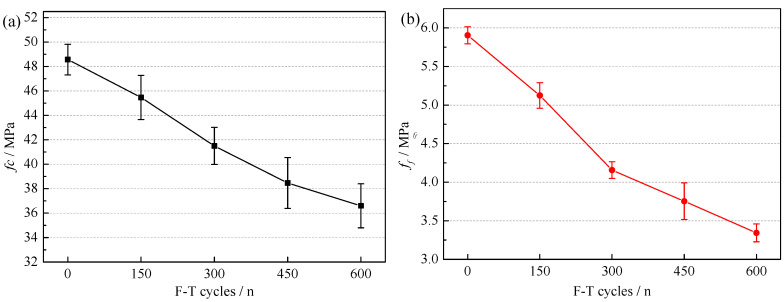
The variations of *f_c_* (**a**) and *f_f_* (**b**) of NAC after F-T cycles.

The mechanical properties of NAC after F-T cycles are shown in Figure 4. The *f_c_* and *f_f_* of NAC were both decreased after F-T cycles. The *f_c_* of NAC-600 was 24.64% lower than that of NAC-0, while the *f_f_* was 43.37% lower than that of NAC-0. This indicated that the *f_f_* was more sensitive to F-T damage than *f_c_*. The strength decreased with the number of F-T cycles, which indicated more F-T damage produced in the NAC. This inevitably affects the physical properties of RCA after concrete crushing [37].

#### 3.1.2. MLR and RDME of NAC

**Figure 5:**
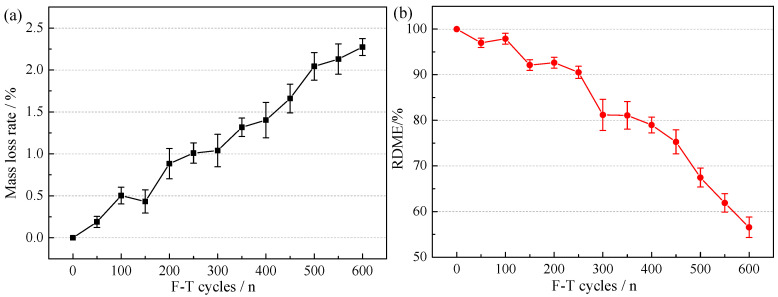
The variations of MLR (**a**) and RDME (**b**) of NAC after F-T cycles.

The variations of MLR and RDME of NAC after F-T cycles are illustrated in Figure 5a,b, respectively. In Figure 5, the MLR of NAC increased gradually with the increasing F-T cycles, and the RDME decreased with the increasing F-T cycles. Although the MLR of NAC was less than 5% after F-T 600 cycles, the RDME of NAC was less than 60%. It indicated that the surface and internal structure of NAC were damaged to varying degrees. The water frozen at a low temperature of NAC internal volume increased by about 9%, which exerted expansion stress on the capillary walls inside concrete [38]. When the swelling stress is greater than the tensile strength of the capillary wall, microcracks and internal structure damage occurred, leading to concrete degeneration, fragile until the fracture collapse. Therefore, it was more reasonable to quantify F-T damage by RDME.

#### 3.1.3. F-T Damage of NAC

**Figure 6:**
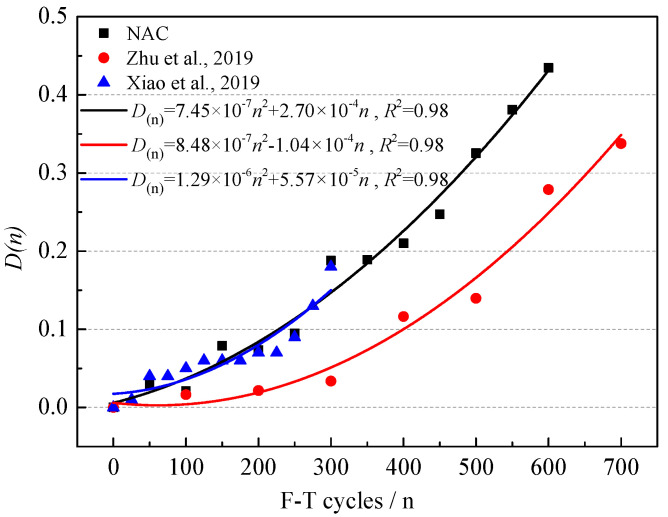
The variation of D_(n)_ of NAC during F-T cycles [18,42].

According to Formula (9), the F-T damage D_(*n*)_ under different F-T cycles was obtained [39]. The variation of F-T damage of NAC is shown in Figure 6. The F-T damage increased rapidly with the F-T cycles, which indicated that the internal structure of NAC was seriously damaged. A quadratic function is a common fitting method between F-T damage and the number of F-T cycles [40,41,42]. The fitting formula is shown in Formula (10).

(9) $$D_{\left(n\right)}=1-\frac{E_{n}}{E_{0}}=1-RDME$$

where, *D*_(*n*)_ represents the F-T damage value of concrete after *n* F-T cycles, *E*_0_ represents the initial RDME of concrete, and *E_n_* represents the RDME of concrete after *n* F-T cycles.

(10) $$D_{\left(n\right)}=7.45\times 10^{-7}n^{2}+2.70\times 10^{-4}n\;R^{2}=0.98\;$$

Frost heave stress caused by F-T cycles repeatedly acted on the interior of NAC. When the stresses exceeded the tensile strength limit of NAC, cracks occurred in the internal structure of NAC, and cracks further expanded until F-T failure [39].

### 3.2. The RCA Properties under Different F-T Damage

#### 3.2.1. Particle Size Variation of RCA

**Figure 7:**
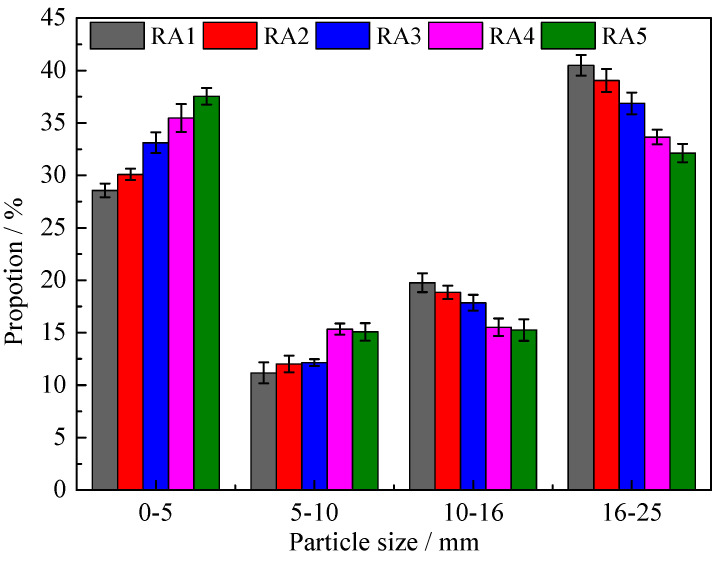
The particle size proportions of RA-*i*.

The particle size proportions of recycled aggregate (RA) are shown in Figure 7. Each RA was randomly selected for screening three times, and at least 5 kg was weighed each time. The average value and error were calculated. The RA-*i* was obtained from the NAC every F-T 150 cycles until F-T failure. As the F-T cycles of NAC increased, the particle size proportion of 0–5 mm of RA increased and the proportion of RCA with particle size greater than 5 mm decreased.

The pick-up rate of RCA is the ratio of the mass of RCA with a particle size above 5 mm to the mass of total RA, as shown in Formula (11). The pick-up rate of RCA was reduced with the development of F-T cycles of NAC. The pick-up rate of RCA1, RCA2, RCA3, RCA4 and RCA5 were 71.43%, 69.91%, 66.88%, 64.53% and 62.46% respectively. The pick-up rate of RCA5 was 12.56% lower than RCA1, which indicated that the F-T damage had a negative effect on the pick-up rate of RCA.

(11) $$P_{RCA}=\frac{M_{RCA}}{M_{RA}}\times 100\%$$

where, *P_RCA_* represents the pick-up rate of RCA; *M_RCA_* represents the mass of RCA; *M_RA_* represents the mass of total RA.

The F-T damage resulted in the decrease of mechanical properties of NAC, which leads to more mortar peeling during the crushing process of NAC. With the increase of F-T cycles of NAC, the RCA particle size decreased and the number of mortar particles increased, which was bound to affect the physical properties of RCA [11].

#### 3.2.2. Physical Properties Variation of RCA

**Figure 8:**
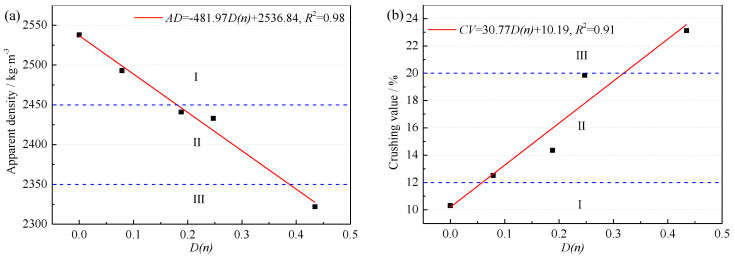

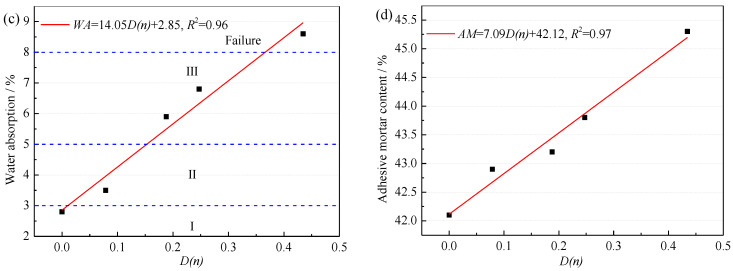
Variation law of apparent density (**a**) crushing value (**b**) water absorption (**c**) and adhesive mortar content (**d**) of RCA with F-T damage of parent concrete.

Figure 8 shows the variation law of physical properties of RCA with F-T damage of parent concrete. As the increase of F-T damage, the apparent density (Figure 8a) of RCA linearly decreased, and the crushing value (Figure 8b) and water absorption (Figure 8c) increased. The apparent density and crushing value of RCA5 obtained from NAC after F-T failure still meet the requirements of Class III RCA in GB/T 25177(2010) [34]. However, the water absorption of RCA5 was greater than 8%, which cannot meet the water absorption requirement of Class III RCA; thus, RCA5 was not recommended for structural concrete.

Pores and microcracks were generated in the F-T cycles of NAC and further expanded due to the external loads in the crushing process. It can be inherited from RCA, leading to the reduction of physical properties of RCA. Meanwhile, it can be learned from the previous Section 3.2.1 that more small RCA particles were produced in larger F-T damage of NAC. The larger specific surface area of RCA resulted in more water absorption of RCA. It was also an important reason why the water absorption cannot meet the requirement of RCA for structural concrete.

The F-T damage had the greatest impact on water absorption index by comparing various physical properties. Therefore, the fitting equation of water absorption and F-T damage of NAC was established, as shown in Formula (12). GB/T 25177(2010) [34] specifies the maximum water absorption of RCA for structural concrete is 8%. This value can be substituted into Formula (12), and *D*_(*n*)_ can be inversely calculated as 0.367. It suggested that the F-T damage threshold of RCA as structural concrete coarse aggregate is 0.367.

(12) $$WA=14.05D_{\left(n\right)}+2.85,\;R^{2}=0.96\;$$

In Figure 8d, the adhesive mortar content of RCA increased with the accumulation of F-T damage in NAC. The components of RCA were mixed with separated mortar particles. The more F-T damage of NAC, the smaller size mortar particles existed in RCA. These mortar particles were difficult to be completely distinguished and were easier to adhere to RCA [43], which led to higher adhesive mortar content in RCA.

### 3.3. Performance of RAC during F-T Cycles

#### 3.3.1. Mechanical Properties of RAC

**Figure 9:**
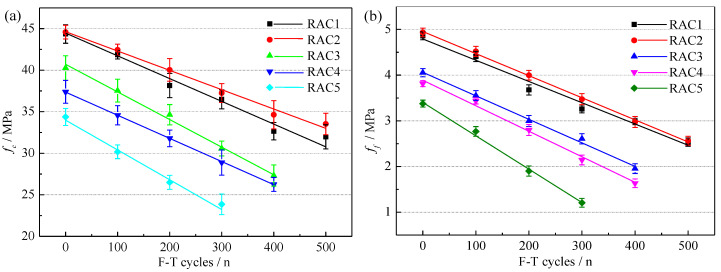
The variations of *f_c_* (**a**) and *f_f_* (**b**) of RAC-*i*.

Figure 9 shows the variations of *f_c_* and *f_f_* of RAC-*i*. It can be found that the *f_c_* and *f_f_* of RACs decreased linearly with the increase of F-T cycles. In Figure 9, it is expected that RAC5 had the worst mechanical properties. It was caused by the difference in the physical properties of RCA.

On the one hand, the F-T damage caused more holes and microcracks to appear in the adhesive mortar, weaken the ITZ, and had an adverse impact on the strength of RAC [44]. On the other hand, for the worse physical properties of RCA, more total water consumption was required to prepare RAC, which led to a higher total water-cement ratio and the reduction of mechanical properties. Unexpectedly, RAC2 had better mechanical properties than RAC1, the possible reasons are as follows: RCA2 further absorbed water during the preparation of RAC2, resulting in the actual water binder ratio of RAC2 being less than RAC1 [45]; Besides, the water consumption of RAC1 was small, which led to insufficient hydration of RAC1 and delayed the hydration speed [46]. In addition, the 28-d *f_c_* of RAC2 was 29.71% higher than that of RAC5, while the 28-d *f_f_* of RAC2 was 45.86% higher than that of RAC5, which indicated that the F-T damage of NAC had a more significant adverse impact on the *f_f_* of RAC.

#### 3.3.2. MLR and RDME of RAC

**Figure 10:**
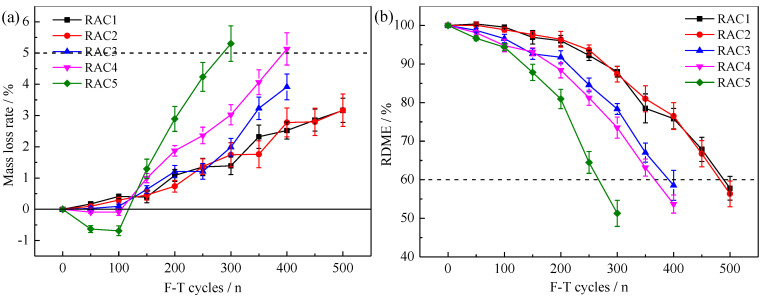
The variations of MLR (**a**) and RDME (**b**) of RAC-*i*.

The variations of MLR and RDME of RAC-*i* are shown in Figure 10. F-T failure occurred in RAC1, RAC2, RAC3, RAC4 and RAC5 after F-T 500, 500, 400, 400 and 300 cycles respectively. Obviously, RAC1 and RAC2 had better frost resistance, followed by RAC3 and RAC4, and RAC5 was the worst. The F-T damage of parent concrete had an adverse impact on the frost resistance of RAC. When the F-T damage of parent concrete was more serious, the frost resistance of RAC was worse.

#### 3.3.3. Service Life Analysis

**Figure 11:**
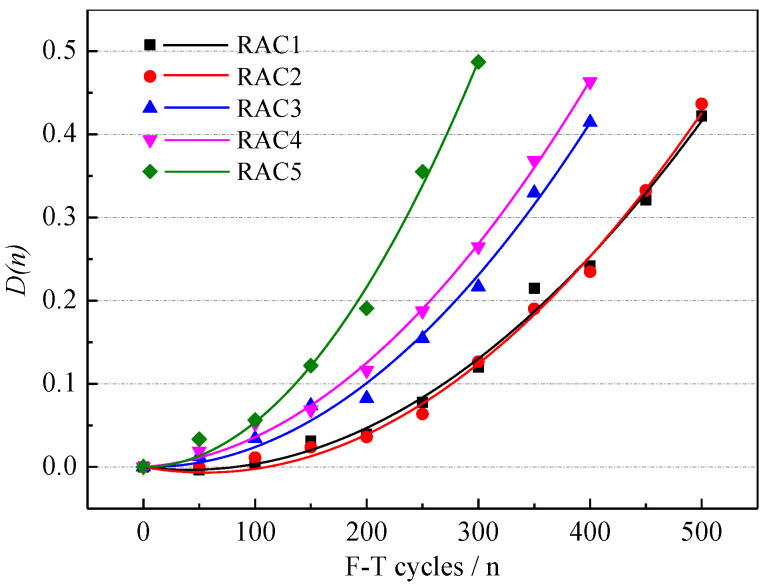
The variations of F-T damage of RAC-*i*.

The F-T damage of RAC was calculated according to formula (9). The variations of F-T damage of RAC-*i* are shown in Figure 11. According to the curve relationship model between F-T damage and F-T cycles [40], the fitting formula of F-T damage and laboratory F-T cycles of five types of RACs were obtained, as shown in Table 4. When the RDME of RAC reduced to 60%, F-T failure of RAC can be considered according to GB/T 50082(2009) [33]. The F-T damage can be calculated as 0.4 by formula (9). The corresponding F-T cycles were obtained in the laboratory.

One rapid F-T cycle in the laboratory was equivalent to F-T 12 cycles in the natural environment [39]. The conversion relationship between the F-T cycles under different service lives in natural F-T environments and the F-T cycles in laboratories are shown in formula (13).

(13) $$t=\frac{12\cdot N}{y}$$

where, *N* represents the laboratory F-T cycles (*n*); *y* represents the average F-T cycles per year; *t* represents the service life (a) in a natural F-T environment.

The cold region was represented by North China, and its annual average F-T cycle was 84 cycles [39]. According to formula (13), the actual service life of five kinds of RACs in cold regions can be obtained, and the results as shown in Table 4.

It can be found from Table 4 that the RAC1, RAC2, RAC3, RAC4, and RAC5 could serve 70, 69, 56, 52, and 38 years in a natural F-T environment, respectively, while the NAC could serve 81 years. The service life of RAC was lower than that of NAC. The RAC produced from parent concrete without F-T failure can serve 50 years in cold regions, while that with F-T failure can only serve 30 years.

#### 3.3.4. Porosity

**Figure 12:**
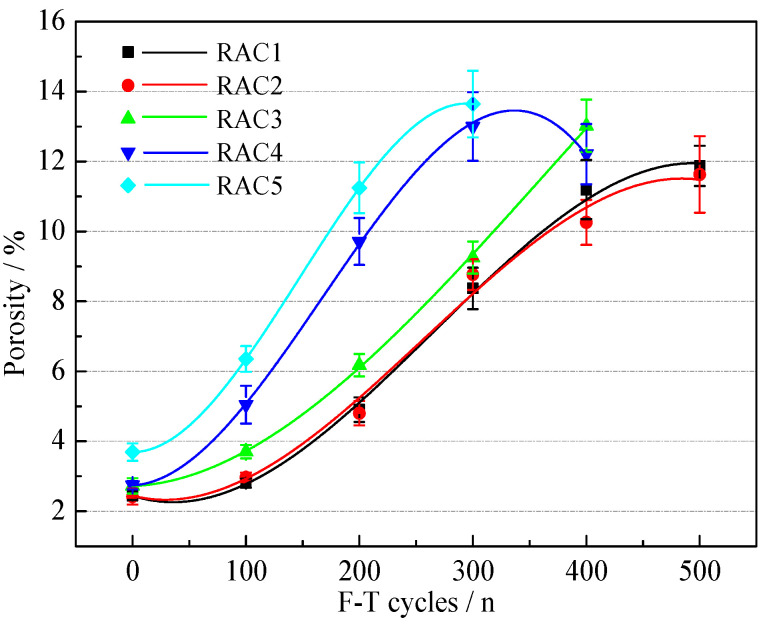
The variations of porosity of RAC-*i*.

The variations of the porosity of RAC are shown in Figure 12. The porosity of all five RACs increased with increasing F-T cycles, which was similar to previous studies [47]. In the early F-T period, the porosity of RACs increased slowly. The addition of AEA optimized the pore structure of RAC, which can relieve the expansion stress caused by free water freezing in the early F-T period, and thus effectively resist the destruction of pore structure caused by F-T damage [21]. In the middle F-T period, with the development of F-T cycles, harmless pores gradually became harmful pores, resulting in a rapid increase in porosity, which indicated that the internal structure of RACs was seriously damaged. In the late F-T period, the growth of porosity became slow, and the porosity of RAC4 showed significant negative growth. This was due to the collapse of the pore wall caused by the expansion of the large pores to the limit, and the peeling mortar-filled part of the large pores [48].

Generally, as shown in Figure 12, the porosity of RAC1 and RAC2 grew slowly, followed by RAC3, while RAC4 and RAC5 grew quickly. The physical properties of RCA and the water-binder ratio of RAC have an important effect on the porosity of RAC [49]. RCA5 had the worst physical properties and required the most additional water for the preparation of RAC5, so its internal structure was poor and its ability to resist F-T damage was weak.

### 3.4. Microstructure

**Figure 13:**
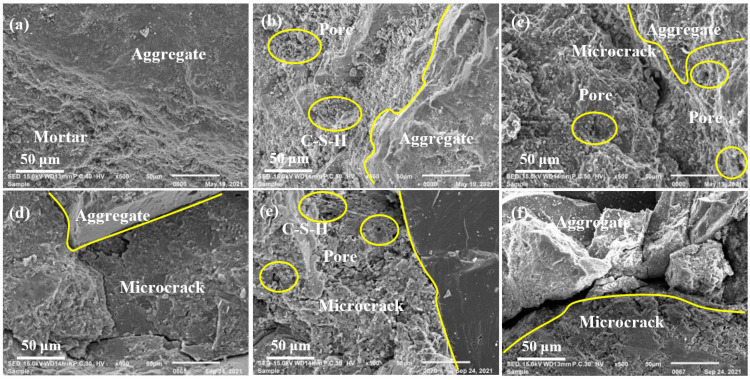
Micro morphology (**a**) NAC-0 (**b**) NAC-300 (**c**) NAC-600 (**d**) RAC1 (**e**) RAC3 (**f**) RAC5.

The micro morphologies of NAC-0, NAC-300, and NAC-600 are illustrated in Figure 13a–c. In Figure 13a, the mortar of NAC-0 was closely connected with aggregate, and the microstructure was complete. After 300 F-T cycles, numerous pores appeared (Figure 13b). The ITZ between mortar and aggregate was easily broken, although the adhesive mortar could hydrate continuously [50]. After 600 F-T cycles, wide microcracks appeared (Figure 13c), which caused mortar particles to spall and the cohesion decreasing. At this time, F-T failure of NAC had occurred.

The micro morphology of RAC1, RAC3, and RAC5 after F-T 300 cycles are illustrated in Figure 13d–f. Although microcracks appeared in the ITZ of RAC1 (Figure 13d), the microcracks were relatively narrow, and the mortar was still relatively dense, which indicated that RAC1 still had the potential to resist F-T damage. In Figure 13e, although rod-like and fibrous C-S-H gel was observed in RAC3, microcracks perpendicular to the ITZ appeared and the internal structure of RAC3 had become broken and porous. In Figure 13f, the microcrack width of RAC5 increased obviously, and the microstructure was worst, which caused the F-T failure. The F-T damage degree of NAC had an important impact on the microstructure of RAC, and then affect the frost resistance of RAC. The more serious the F-T damage was to NAC, the worse the microstructure of RAC and the weaker its ability to resist F-T damage.

**Figure 14:**
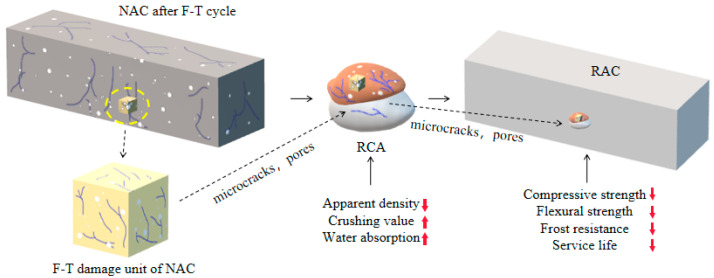
Effect mechanism of NAC F-T damage on properties of RAC.

The influence mechanism of NAC F-T damage on the mechanical and frost resistance of RAC is shown in Figure 14. Plenty of pores and microcracks were produced after NAC F-T cycling, and some parts of NAC could be regarded as microelements of F-T damage. After the broken of NAC, the damaged microelement dispersed in the adhesive mortar of RCA, resulting in the decline of the physical properties. When RCA was used to prepare RAC, these microelements were transferred again, which caused worse internal structure in RAC, and ultimately led to the macroscopic properties of RAC decreasing. There was no doubt that the adhesive mortar of RCA played an important role in the intergenerational transfer of NAC F-T damage.

### 3.5. Sustainability Analysis

**Figure 15:**
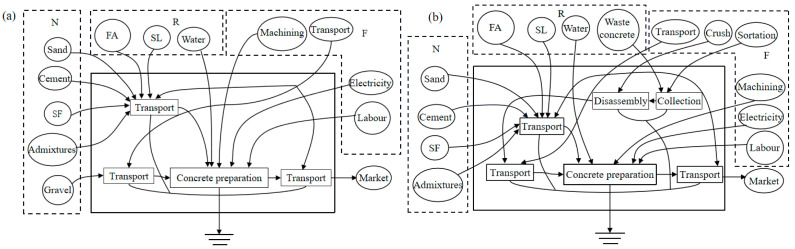
The flow mode of energy in concrete production is (**a**) NAC; (**b**) RAC.

The emergy flow modes of NAC and RAC production are illustrated in Figure 15. The emergy evaluation form of NAC is shown in Table 5. The input/output amount was calculated according to the materials required to produce 1 m^3^ concrete. Due to space limitation, Table 6 shows the emergy evaluation form for the same items of RACi, and the different items are shown in Table 7.

The basic emergy flow and emergy evaluation index of concrete can be calculated according to Table 6 and Table 7, and the results are shown in Table 8. The EIR and EYR of NAC were smaller than RACi, indicating the higher productivity and output of the RACi production mode. The marginal output of the NAC production mode was small and the output efficiency was low. Besides, the ELR of the RACi production mode was about 5% of that of NAC. Obviously, the environmental burden of the RACi production mode was much lower than that of NAC. The ESI of NAC was less than 1, which indicated that the NAC production mode was not sustainable.

The ESI of RACi was greater than 1, RACi production mode had better sustainability. Although the RACi production mode increased the investment of social resources, with the integration and upgrading of the construction waste treatment industrial chain [26], the gradual improvement of RAC performance theory and the continuous amelioration of RAC construction technology, the sustainable development ability of RAC was bound to be gradually improved. It was worth noting that RAC5 had the smallest EYR and ESI and the largest EIR and ELR, which indicated that the reuse of waste concrete after F-T failure required higher economic input, higher environment load, lower output efficiency, and sustainability.

To sum up, the performance, environmental load, and economic benefit of RAC prepared by using waste concrete after F-T failure are inferior to that of waste concrete without F-T failure. The waste concrete after F-T failure needed to be modified to improve RAC performance, which might cause a change in the emergy evaluation index. By developing a new RAC mix proportion, or further improving the modification technology of RCA and optimizing the proportion of various emergy inputs, the social resource input of RAC production can be reduced, the output efficiency of RAC can be enhanced, and then the sustainable application potential of waste concrete can be improved.

## 4. Conclusions

In this paper, the effects of NAC freeze-thaw damage on the properties of RCA, the mechanical properties, and frost resistance of RAC were studied; besides, the sustainable utilization potential of waste NAC in a freeze-thaw environment was comprehensively evaluated from three aspects of performance, environmental load, and economic benefits. The specific conclusions are as follows.

(1) As the number of freeze-thaw cycles of parent concrete increased, the particle size proportion of 0–5 mm of recycled aggregate increased and the pick-up rate of RCA reduced. The freeze-thaw damage of parent concrete adversely affected the physical properties of RCA. With the increase of freeze-thaw damage, the apparent density of RCA linearly decreased, while the crushing value and water absorption of RCA increased. The RCA5 produced from parent concrete suffered freeze-thaw failure and was not recommended for structural concrete as the water absorption was above 8%. The RCA could be used as structural concrete coarse aggregate when the freeze-thaw damage of NAC was smaller than 0.367.
(2) The freeze-thaw damage of parent concrete also had an adverse impact on the mechanical properties and frost resistance of RAC. The frost resistance of RAC obtained from parent concrete with larger freeze-thaw damage was worse. The service life of RAC1, RAC2, RAC3, and RAC4 in cold regions can reach 70, 69, 56, and 52 years respectively, while that of RAC5 prepared from parent concrete with freeze-thaw failure was only 38 years. The weaker interfacial transition zone and porosity of RAC were the fundamental reason for the weakening of the properties of RAC.
(3) The adhesive mortar of RCA played an important role in the intergenerational transfer of parent concrete freeze-thaw damage. The freeze-thaw damaged microelement dispersed in the adhesive mortar of RCA, resulting in the decline of the physical properties of RCA. When RCA was used to prepare RAC, these microelements were transferred again, which led to a decrease in the macroscopic mechanical properties and frost resistance of RAC.
(4) Emergy analysis showed that the RAC production mode had more sustainable application potential than NAC. The reuse of waste concrete after freeze-thaw failure required higher economic input, higher environmental load, lower output efficiency, and sustainability. The performance, environmental load and economic benefit of RAC prepared by using waste concrete after freeze-thaw failure were inferior to that of waste concrete without freeze-thaw failure.

The limitation of this study is that RAC was prepared at a 100% replacement rate, and preparations under different replacement rates were not involved. Besides, the emergy conversion rate is different under different spatial and temporal conditions, which will have an uncertain impact on the research results. With the application of emergy theory in RAC sustainable production, the emergy theory system needs more basic data to be improved. The study enriches the sustainability analysis database of recycled aggregate concrete and provides a theoretical basis for the preparation of standards. Under the background of vigorously advocating green and low-carbon economy all over the world, the sustainable application potential of modified RCA to produce RAC by green and low-carbon methods will be a hot topic in future research.

## Figures and Tables

**Table 1:** The physical properties of NCA and RCAs.

Category	Apparent Density (kg·m^−3^)	Bulk Density (kg·m^−3^)	Crushing Value (%)	Water Absorption (%)	Particle Size (mm)	Adhesive Mortar Content (%)
NCA	2654	1518	5.1	0.3	5–25	-
RCA1	2538	1449	10.31	2.8		42.1
RCA2	2493	1437	12.52	3.5		42.9
RCA3	2441	1413	14.36	5.9		43.2
RCA4	2433	1405	19.85	6.8		43.8
RCA5	2322	1359	23.12	8.6		45.3

**Table 2:** Mix proportions and slumps.

Group	Mix Proportions (kg·m^−3^)										Slump (mm)
	NCA	RCA	Sand	Cement	SF	FA	SL	PCA	AEA	Total Water Content	
NAC	1052	-	665	300	23	92	46	2.3	0.28	176	185
RAC1	-	1003	667	300	23	92	46	2.3	0.28	189	181
RAC2	-	999	653	300	23	92	46	2.3	0.28	194	173
RAC3	-	984	646	300	23	92	46	2.3	0.28	203	175
RAC4	-	978	650	300	23	92	46	2.3	0.28	207	168
RAC5	-	951	630	300	23	92	46	2.3	0.28	219	170

**Table 3:** The corresponding relationship among NAC, RCA and RAC.

Type	Aggregate	Source
RAC1	RCA1	NAC-0
RAC2	RCA2	NAC-150
RAC3	RCA3	NAC-300
RAC4	RCA4	NAC-450
RAC5	RCA5	NAC-600

Note: NAC-i represents the NAC after F-T *i* cycles.

**Table 4:** The fitting formula, laboratory F-T cycles, and service life in cold regions.

Type	Fitting Formula	*R* ^2^	Laboratory F-T Cycles (n)	Service Life (a)
RAC1	*D*(*n*) = 1.99 × 10^−6^*n*^2^ − 1.64 × 10^−4^*n*	0.993	491	70
RAC2	*D*(*n*) = 2.19 × 10^−6^*n*^2^ − 2.42 × 10^−4^*n*	0.995	486	69
RAC3	*D*(*n*) = 2.65 × 10^−6^*n*^2^ − 2.56 × 10^−5^*n*	0.992	393	56
RAC4	*D*(*n*) = 2.68 × 10^−6^*n*^2^ + 9.17 × 10^−5^*n*	0.997	369	52
RAC5	*D*(*n*) = 5.44 × 10^−6^*n*^2^ − 5.76 × 10^−6^*n*	0.992	271	38
NAC	*D*(*n*) = 7.45 × 10^−7^*n*^2^ + 2.70 × 10^−4^*n*	0.983	573	81

**Table 5:** Emergy evaluation form of NAC.

No.	Item	Units	Input/Output Amount	Unit Emergy Values (sej/unit)	Solar Emergy (sej)	Ref.
Non-renewable resources (N)						
1	Gravel	g	1.05 × 10^6^	2.46 × 10^9^	2.58 × 10^15^	[51]
2	Sand	g	6.65 × 10^5^	2.46 × 10^9^	1.64 × 10^15^	[51]
3	Cement	g	3.00 × 10^5^	1.73 × 10^9^	5.19 × 10^14^	[26]
4	SF	g	2.30 × 10^4^	1.00 × 10^9^	2.30 × 10^13^	[25]
5	PCA	g	2.30 × 10^3^	1.68 × 10^9^	3.86 × 10^12^	[25]
6	AEA	g	2.80 × 10^2^	1.68 × 10^9^	4.70 × 10^11^	[25]
Renewable resources (R)						
7	Water	g	1.76 × 10^5^	1.26 × 10^6^	2.22 × 10^11^	[52]
8	FA	g	9.20 × 10^4^	1.68 × 10^9^	1.55 × 10^14^	[53]
9	SL	g	4.60 × 10^4^	1.68 × 10^9^	7.73 × 10^13^	[53]
Non-raw material inputs (F)						
10	Electricity	kJ	2.38 × 10^4^	1.59 × 10^8^	3.78 × 10^12^	[54]
11	Machining	J	9.50 × 10^2^	9.21 × 10^9^	8.75 × 10^12^	[55]
12	Transport	t×m	1.20 × 10^3^	7.61 × 10^11^	9.13 × 10^14^	[51]
13	Labour	$	5.58 × 10^2^	1.06 × 10^11^	5.91 × 10^13^	[56]
Yield emergy flow (Y)						
14	NAC	g	2.50 × 10^6^	1.81 × 10^9^	4.53 × 10^15^	[55]

**Table 6:** Emergy evaluation form for the same items of RACi.

No.	Item	Units	Input/Output Amount	Unit Emergy Values (sej/unit)	Solar Emergy (sej)	Ref.
Non-renewable resources (N)						
1	Sand	g	a	2.46 × 10^9^	-	[51]
2	Cement	g	3.00 × 10^5^	1.73 × 10^9^	5.19 × 10^14^	[26]
3	SF	g	2.30 × 10^4^	1.00 × 10^9^	2.30 × 10^13^	[25]
4	PCA	g	2.30 × 10^3^	1.68 × 10^9^	3.86 × 10^12^	[25]
5	AEA	g	2.80 × 10^2^	1.68 × 10^9^	4.70 × 10^11^	[25]
Renewable resources (R)						
6	RCAi	g	b	2.46 × 10^9^	-	[51]
7	Water	g	c	1.26 × 10^6^	-	[52]
8	FA	g	9.20 × 10^4^	1.68 × 10^9^	1.55 × 10^14^	[53]
9	SL	g	4.60 × 10^4^	1.68 × 10^9^	7.73 × 10^13^	[53]
Non-raw material inputs (F)						
10	Electricity	kJ	2.38 × 10^4^	1.59 × 10^8^	3.78 × 10^12^	[54]
11	Collection	g	b	5.11 × 10^7^	-	[25]
12	Disassembly	g	b	6.75 × 10^6^	-	[25]
13	Machining	J	9.50 × 10^2^	9.21 × 10^9^	8.75 × 10^12^	[55]
14	Transport	t×m	1.20 × 10^3^	7.61 × 10^11^	9.13 × 10^14^	[51]
15	Labour	$	5.58 × 10^2^	1.06 × 10^11^	5.91 × 10^13^	[56]
Yield emergy flow (Y)						
16	RACi	g	d	2.18 × 10^9^	-	[55]

Notes: a represents the input amount of sand in RACi. b represents the input amount of RCAi, Collection and Disassembly in RACi. c represents the input amount of Water in RACi. d represents the output amount of RACi.

**Table 7:** Emergy evaluation form for different items of RACi.

Type	Item											
	Sand		RCAi		Water		Collection		Disassembly		Yield Emergy Flow	
	I (×10^5^ g)	SE (×10^15^ sej)	I (×10^5^ g)	SE (×10^15^ sej)	I (×10^5^ g)	SE (×10^11^ sej)	I (×10^5^ g)	SE (×10^13^ sej)	I (×10^6^ g)	SE (×10^12^ sej)	Y (×10^6^ g)	SE (×10^15^ sej)
RAC1	6.67	1.64	10.03	2.47	1.89	2.38	10.03	5.13	10.03	6.77	2.42	5.28
RAC2	6.53	1.61	9.99	2.46	1.94	2.44	9.99	5.10	9.99	6.74	2.38	5.19
RAC3	6.46	1.59	9.84	2.42	2.03	2.56	9.84	5.03	9.84	6.64	2.36	5.14
RAC4	6.50	1.60	9.78	2.41	2.07	2.61	9.78	5.00	9.78	6.60	2.34	5.10
RAC5	6.30	1.55	9.51	2.34	2.19	2.76	9.51	4.86	9.51	6.42	2.30	5.01

**Table 8:** Emergy indexes for sustainable analysis of NAC and RACi.

Indexes	Type					
	NAC	RAC1	RAC2	RAC3	RAC4	RAC5
Basic emergy flow (×10^14^ sej)						
N	47.65	21.87	21.53	21.35	21.45	20.96
R	2.32	26.99	26.90	26.53	26.38	25.72
F	9.85	10.43	10.43	10.42	10.41	10.40
Y	45.30	52.76	51.88	51.45	51.01	50.14
Emergy evaluation index						
EIR	0.197	0.213	0.215	0.217	0.218	0.223
EYR	4.594	5.059	4.976	4.938	4.898	4.822
ELR	24.78	1.197	1.188	1.198	1.208	1.219
ESI	0.185	4.228	4.188	4.123	4.055	3.954

## Data Availability

The data used to support the findings of this study are available from the corresponding author upon request.
